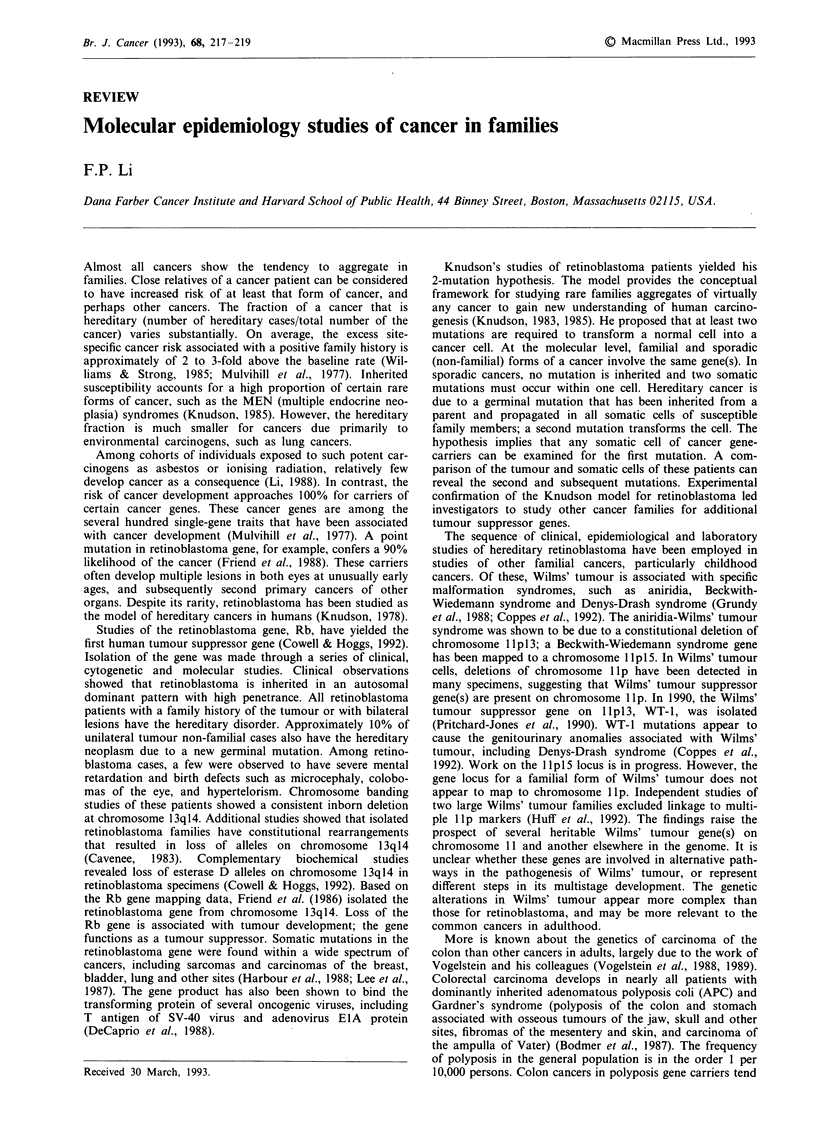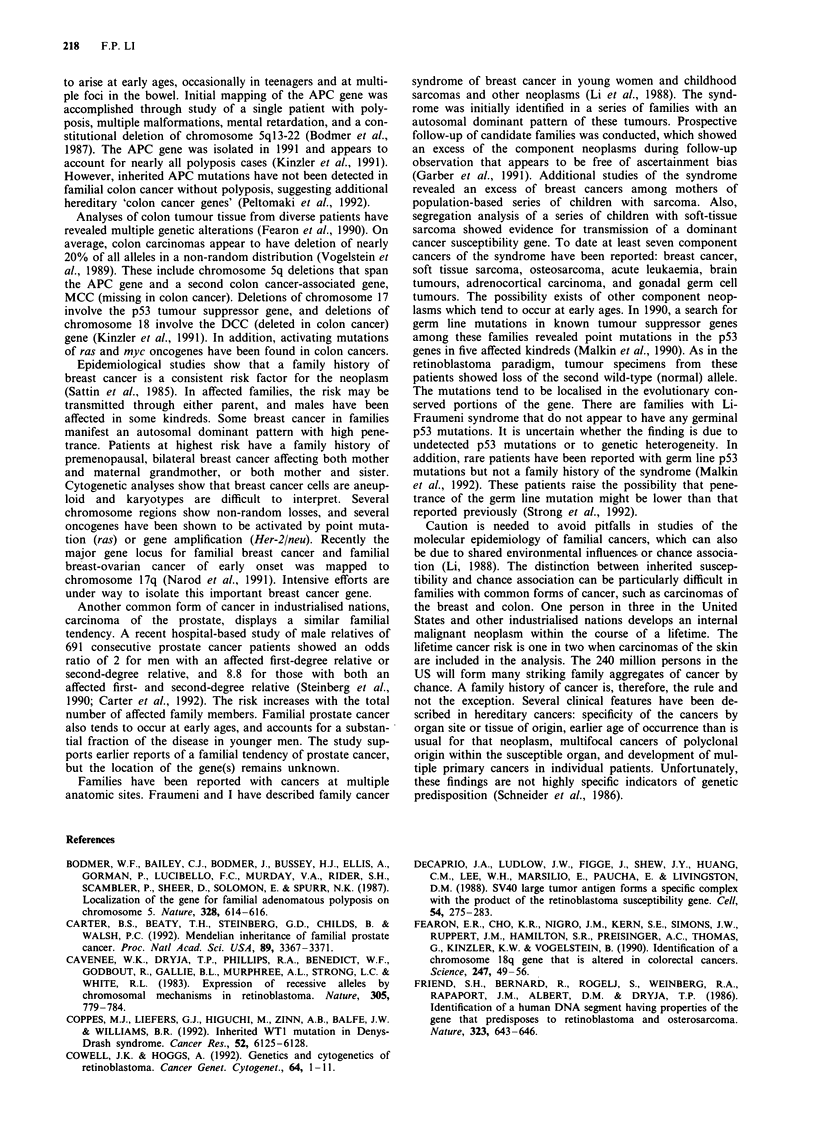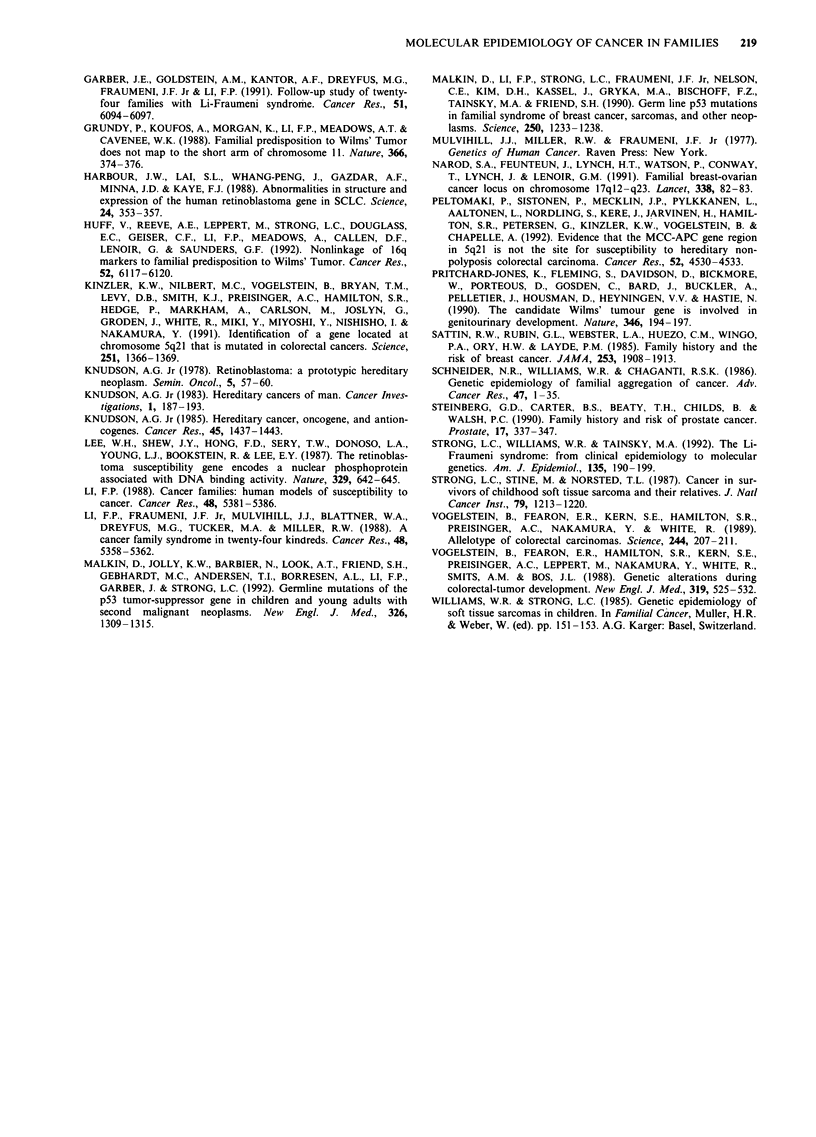# Molecular epidemiology studies of cancer in families.

**DOI:** 10.1038/bjc.1993.318

**Published:** 1993-08

**Authors:** F. P. Li

**Affiliations:** Dana Farber Cancer Institute, Boston, Massachusetts.


Br. J. Cancer (1993), 68, 217–219 © Macmillan Press Ltd., 1993

REVIEW

### Molecular epidemiology studies of cancer in families

F.P. Li

*Dana Farber Cancer Institute and Harvard School of Public Health, 44 Binney Street, Boston, Massachusetts 02115, USA.*

Almost all cancers show the tendency to aggregate in families. Close relatives of a cancer patient can be considered to have increased risk of at least that form of cancer, and perhaps other cancers. The fraction of a cancer that is hereditary (number of hereditary cases/total number of the cancer) varies substantially. On average, the excess site-specific cancer risk associated with a positive family history is approximately of 2 to 3-fold above the baseline rate (Williams & Strong, 1985; Mulvihill *et al.*, 1977). Inherited susceptibility accounts for a high proportion of certain rare forms of cancer, such as the MEN (multiple endocrine neoplasia) syndromes (Knudson, 1985). However, the hereditary fraction is much smaller for cancers due primarily to environmental carcinogens, such as lung cancers.

Among cohorts of individuals exposed to such potent carcinogens as asbestos or ionising radiation, relatively few develop cancer as a consequence (Li, 1988). In contrast, the risk of cancer development approaches 100% for carriers of certain cancer genes. These cancer genes are among the several hundred single-gene traits that have been associated with cancer development (Mulvihill *et al.*, 1977). A point mutation in retinoblastoma gene, for example, confers a 90% likelihood of the cancer (Friend *et al.*, 1988). These carriers often develop multiple lesions in both eyes at unusually early ages, and subsequently second primary cancers of other organs. Despite its rarity, retinoblastoma has been studied as the model of hereditary cancers in humans (Knudson, 1978).

Studies of the retinoblastoma gene, Rb, have yielded the first human tumour suppressor gene (Cowell & Hoggs, 1992). Isolation of the gene was made through a series of clinical, cytogenetic and molecular studies. Clinical observations showed that retinoblastoma is inherited in an autosomal dominant pattern with high penetrance. All retinoblastoma patients with a family history of the tumour or with bilateral lesions have the hereditary disorder. Approximately 10% of unilateral tumour non-familial cases also have the hereditary neoplasm due to a new germinal mutation. Among retinoblastoma cases, a few were observed to have severe mental retardation and birth defects such as microcephaly, colobomas of the eye, and hypertelorism. Chromosome banding studies of these patients showed a consistent inborn deletion at chromosome 13q14. Additional studies showed that isolated retinoblastoma families have constitutional rearrangements that resulted in loss of alleles on chromosome 13q14 (Cavenee, 1983). Complementary biochemical studies revealed loss of esterase D alleles on chromosome 13q14 in retinoblastoma specimens (Cowell & Hoggs, 1992). Based on the Rb gene mapping data, Friend *et al.* (1986) isolated the retinoblastoma gene from chromosome 13q14. Loss of the Rb gene is associated with tumour development; the gene functions as a tumour suppressor. Somatic mutations in the retinoblastoma gene were found within a wide spectrum of cancers, including sarcomas and carcinomas of the breast, bladder, lung and other sites (Harbour *et al.*, 1988; Lee *et al.*, 1987). The gene product has also been shown to bind the transforming protein of several oncogenic viruses, including T antigen of SV-40 virus and adenovirus E1A protein (DeCaprio *et al.*, 1988).

Received 30 March, 1993.

Knudson's studies of retinoblastoma patients yielded his 2-mutation hypothesis. The model provides the conceptual framework for studying rare families aggregates of virtually any cancer to gain new understanding of human carcinogenesis (Knudson, 1983, 1985). He proposed that at least two mutations are required to transform a normal cell into a cancer cell. At the molecular level, familial and sporadic (non-familial) forms of a cancer involve the same gene(s). In sporadic cancers, no mutation is inherited and two somatic mutations must occur within one cell. Hereditary cancer is due to a germinal mutation that has been inherited from a parent and propagated in all somatic cells of susceptible family members; a second mutation transforms the cell. The hypothesis implies that any somatic cell of cancer gene-carriers can be examined for the first mutation. A comparison of the tumour and somatic cells of these patients can reveal the second and subsequent mutations. Experimental confirmation of the Knudson model for retinoblastoma led investigators to study other cancer families for additional tumour suppressor genes.

The sequence of clinical, epidemiological and laboratory studies of hereditary retinoblastoma have been employed in studies of other familial cancers, particularly childhood cancers. Of these, Wilms' tumour is associated with specific malformation syndromes, such as aniridia, Beckwith-Wiedemann syndrome and Denys-Drash syndrome (Grundy *et al.*, 1988; Coppes *et al.*, 1992). The aniridia-Wilms' tumour syndrome was shown to be due to a constitutional deletion of chromosome 11p13; a Beckwith-Wiedemann syndrome gene has been mapped to a chromosome 11p15. In Wilms' tumour cells, deletions of chromosome 11p have been detected in many specimens, suggesting that Wilms' tumour suppressor gene(s) are present on chromosome 11p. In 1990, the Wilms' tumour suppressor gene on 11p13, WT-1, was isolated (Pritchard-Jones *et al.*, 1990). WT-1 mutations appear to cause the genitourinary anomalies associated with Wilms' tumour, including Denys-Drash syndrome (Coppes *et al.*, 1992). Work on the 11p15 locus is in progress. However, the gene locus for a familial form of Wilms' tumour does not appear to map to chromosome 11p. Independent studies of two large Wilms' tumour families excluded linkage to multiple 11p markers (Huff *et al.*, 1992). The findings raise the prospect of several heritable Wilms' tumour gene(s) on chromosome 11 and another elsewhere in the genome. It is unclear whether these genes are involved in alternative pathways in the pathogenesis of Wilms' tumour, or represent different steps in its multistage development. The genetic alterations in Wilms' tumour appear more complex than those for retinoblastoma, and may be more relevant to the common cancers in adulthood.

More is known about the genetics of carcinoma of the colon than other cancers in adults, largely due to the work of Vogelstein and his colleagues (Vogelstein *et al.*, 1988, 1989). Colorectal carcinoma develops in nearly all patients with dominantly inherited adenomatous polyposis coli (APC) and Gardner's syndrome (polyposis of the colon and stomach associated with osseous tumours of the jaw, skull and other sites, fibromas of the mesentery and skin, and carcinoma of the ampulla of Vater) (Bodmer *et al.*, 1987). The frequency of polyposis in the general population is in the order 1 per 10,000 persons. Colon cancers in polyposis gene carriers tend to arise at early ages, occasionally in teenagers and at multiple foci in the bowel. Initial mapping of the APC gene was accomplished through study of a single patient with polyposis, multiple malformations, mental retardation, and a constitutional deletion of chromosome 5q13-22 (Bodmer *et al.*, 1987). The APC gene was isolated in 1991 and appears to account for nearly all polyposis cases (Kinzler *et al.*, 1991). However, inherited APC mutations have not been detected in familial colon cancer without polyposis, suggesting additional hereditary 'colon cancer genes' (Peltomaki *et al.*, 1992).

Analyses of colon tumour tissue from diverse patients have revealed multiple genetic alterations (Fearon *et al.*, 1990). On average, colon carcinomas appear to have deletion of nearly 20% of all alleles in a non-random distribution (Vogelstein *et al.*, 1989). These include chromosome 5q deletions that span the APC gene and a second colon cancer-associated gene, MCC (missing in colon cancer). Deletions of chromosome 17 involve the p53 tumour suppressor gene, and deletions of chromosome 18 involve the DCC (deleted in colon cancer) gene (Kinzler *et al.*, 1991). In addition, activating mutations of *ras* and *myc* oncogenes have been found in colon cancers.

Epidemiological studies show that a family history of breast cancer is a consistent risk factor for the neoplasm (Sattin *et al.*, 1985). In affected families, the risk may be transmitted through either parent, and males have been affected in some kindreds. Some breast cancer in families manifest an autosomal dominant pattern with high penetrance. Patients at highest risk have a family history of premenopausal, bilateral breast cancer affecting both mother and maternal grandmother, or both mother and sister. Cytogenetic analyses show that breast cancer cells are aneuploid and karyotypes are difficult to interpret. Several chromosome regions show non-random losses, and several oncogenes have been shown to be activated by point mutation (*ras*) or gene amplification (*Her-2/neu*). Recently the major gene locus for familial breast cancer and familial breast-ovarian cancer of early onset was mapped to chromosome 17q (Narod *et al.*, 1991). Intensive efforts are under way to isolate this important breast cancer gene.

Another common form of cancer in industrialised nations, carcinoma of the prostate, displays a similar familial tendency. A recent hospital-based study of male relatives of 691 consecutive prostate cancer patients showed an odds ratio of 2 for men with an affected first-degree relative or second-degree relative, and 8.8 for those with both an affected first- and second-degree relative (Steinberg *et al.*, 1990; Carter *et al.*, 1992). The risk increases with the total number of affected family members. Familial prostate cancer also tends to occur at early ages, and accounts for a substantial fraction of the disease in younger men. The study supports earlier reports of a familial tendency of prostate cancer, but the location of the gene(s) remains unknown.

Families have been reported with cancers at multiple anatomic sites. Fraumeni and I have described family cancer syndrome of breast cancer in young women and childhood sarcomas and other neoplasms (Li *et al.*, 1988). The syndrome was initially identified in a series of families with an autosomal dominant pattern of these tumours. Prospective follow-up of candidate families was conducted, which showed an excess of the component neoplasms during follow-up observation that appears to be free of ascertainment bias (Garber *et al.*, 1991). Additional studies of the syndrome revealed an excess of breast cancers among mothers of population-based series of children with sarcoma. Also, segregation analysis of a series of children with soft-tissue sarcoma showed evidence for transmission of a dominant cancer susceptibility gene. To date at least seven component cancers of the syndrome have been reported: breast cancer, soft tissue sarcoma, osteosarcoma, acute leukaemia, brain tumours, adrenocortical carcinoma, and gonadal germ cell tumours. The possibility exists of other component neoplasms which tend to occur at early ages. In 1990, a search for germ line mutations in known tumour suppressor genes among these families revealed point mutations in the p53 genes in five affected kindreds (Malkin *et al.*, 1990). As in the retinoblastoma paradigm, tumour specimens from these patients showed loss of the second wild-type (normal) allele. The mutations tend to be localised in the evolutionary conserved portions of the gene. There are families with Li-Fraumeni syndrome that do not appear to have any germinal p53 mutations. It is uncertain whether the finding is due to undetected p53 mutations or to genetic heterogeneity. In addition, rare patients have been reported with germ line p53 mutations but not a family history of the syndrome (Malkin *et al.*, 1992). These patients raise the possibility that penetrance of the germ line mutation might be lower than that reported previously (Strong *et al.*, 1992).

Caution is needed to avoid pitfalls in studies of the molecular epidemiology of familial cancers, which can also be due to shared environmental influences or chance association (Li, 1988). The distinction between inherited susceptibility and chance association can be particularly difficult in families with common forms of cancer, such as carcinomas of the breast and colon. One person in three in the United States and other industrialised nations develops an internal malignant neoplasm within the course of a lifetime. The lifetime cancer risk is one in two when carcinomas of the skin are included in the analysis. The 240 million persons in the US will form many striking family aggregates of cancer by chance. A family history of cancer is, therefore, the rule and not the exception. Several clinical features have been described in hereditary cancers: specificity of the cancers by organ site or tissue of origin, earlier age of occurrence than is usual for that neoplasm, multifocal cancers of polyclonal origin within the susceptible organ, and development of multiple primary cancers in individual patients. Unfortunately, these findings are not highly specific indicators of genetic predisposition (Schneider *et al.*, 1986).

### References

BODMER, W.F., BAILEY, C.J., BODMER, J., BUSSEY, H.J., ELLIS, A., GORMAN, P., LUCIBELLO, F.C., MURDAY, V.A., RIDER, S.H., SCAMBLER, P., SHEER, D., SOLOMON, E. & SPURR, N.K. (1987). Localization of the gene for familial adenomatous polyposis on chromosome 5. *Nature*, **328**, 614-616.

CARTER, B.S., BEATY, T.H., STEINBERG, G.D., CHILDS, B. & WALSH, P.C. (1992). Mendelian inheritance of familial prostate cancer. *Proc. Natl Acad. Sci. USA*, **89**, 3367-3371.

CAVENEE, W.K., DRYJA, T.P., PHILLIPS, R.A., BENEDICT, W.F., GODBOUT, R., GALLIE, B.L., MURPHREE, A.L., STRONG, L.C. & WHITE, R.L. (1983). Expression of recessive alleles by chromosomal mechanisms in retinoblastoma. *Nature*, **305**, 779-784.

COPPES, M.J., LIEFERS, G.J., HIGUCHI, M., ZINN, A.B., BALFE, J.W. & WILLIAMS, B.R. (1992). Inherited WT1 mutation in Denys-Drash syndrome. *Cancer Res.*, **52**, 6125-6128.

COWELL, J.K. & HOGGS, A. (1992). Genetics and cytogenetics of retinoblastoma. *Cancer Genet. Cytogenet.*, **64**, 1-11.

DECAPRIO, J.A., LUDLOW, J.W., FIGGE, J., SHEW, J.Y., HUANG, C.M., LEE, W.H., MARSILIO, E., PAUCHA, E. & LIVINGSTON, D.M. (1988). SV40 large tumor antigen forms a specific complex with the product of the retinoblastoma susceptibility gene. *Cell*, **54**, 275-283.

FEARON, E.R., CHO, K.R., NIGRO, J.M., KERN, S.E., SIMONS, J.W., RUPPERT, J.M., HAMILTON, S.R., PREISINGER, A.C., THOMAS, G., KINZLER, K.W. & VOGELSTEIN, B. (1990). Identification of a chromosome 18q gene that is altered in colorectal cancers. *Science*, **247**, 49-56.

FRIEND, S.H., BERNARD, R., ROGELJ, S., WEINBERG, R.A., RAPAPORT, J.M., ALBERT, D.M. & DRYJA, T.P. (1986). Identification of a human DNA segment having properties of the gene that predisposes to retinoblastoma and osterosarcoma. *Nature*, **323**, 643-646.

GARBER, J.E., GOLDSTEIN, A.M., KANTOR, A.F., DREYFUS, M.G., FRAUMENI, J.F. Jr & LI, F.P. (1991). Follow-up study of twenty-four families with Li-Fraumeni syndrome. *Cancer Res.*, **51**, 6094–6097.

GRUNDY, P., KOUFOS, A., MORGAN, K., LI, F.P., MEADOWS, A.T. & CAVENEE, W.K. (1988). Familial predisposition to Wilms' Tumor does not map to the short arm of chromosome 11. *Nature*, **366**, 374–376.

HARBOUR, J.W., LAI, S.L., WHANG-PENG, J., GAZDAR, A.F., MINNA, J.D. & KAYE, F.J. (1988). Abnormalities in structure and expression of the human retinoblastoma gene in SCLC. *Science*, **24**, 353–357.

HUFF, V., REEVE, A.E., LEPPERT, M., STRONG, L.C., DOUGLASS, E.C., GEISER, C.F., LI, F.P., MEADOWS, A., CALLEN, D.F., LENOIR, G. & SAUNDERS, G.F. (1992). Nonlinkage of 16q markers to familial predisposition to Wilms' Tumor. *Cancer Res.*, **52**, 6117–6120.

KINZLER, K.W., NILBERT, M.C., VOGELSTEIN, B., BRYAN, T.M., LEVY, D.B., SMITH, K.J., PREISINGER, A.C., HAMILTON, S.R., HEDGE, P., MARKHAM, A., CARLSON, M., JOSLYN, G., GRODEN, J., WHITE, R., MIKI, Y., MIYOSHI, Y., NISHISHO, I. & NAKAMURA, Y. (1991). Identification of a gene located at chromosome 5q21 that is mutated in colorectal cancers. *Science*, **251**, 1366–1369.

KNUDSON, A.G. Jr (1978). Retinoblastoma: a prototypic hereditary neoplasm. *Semin. Oncol.*, **5**, 57–60.

KNUDSON, A.G. Jr (1983). Hereditary cancers of man. *Cancer Investigations*, **1**, 187–193.

KNUDSON, A.G. Jr (1985). Hereditary cancer, oncogene, and antioncogenes. *Cancer Res.*, **45**, 1437–1443.

LEE, W.H., SHEW, J.Y., HONG, F.D., SERY, T.W., DONOSO, L.A., YOUNG, L.J., BOOKSTEIN, R. & LEE, E.Y. (1987). The retinoblastoma susceptibility gene encodes a nuclear phosphoprotein associated with DNA binding activity. *Nature*, **329**, 642–645.

LI, F.P. (1988). Cancer families: human models of susceptibility to cancer. *Cancer Res.*, **48**, 5381–5386.

LI, F.P., FRAUMENI, J.F. Jr, MULVIHILL, J.J., BLATTNER, W.A., DREYFUS, M.G., TUCKER, M.A. & MILLER, R.W. (1988). A cancer family syndrome in twenty-four kindreds. *Cancer Res.*, **48**, 5358–5362.

MALKIN, D., JOLLY, K.W., BARBIER, N., LOOK, A.T., FRIEND, S.H., GEBHARDT, M.C., ANDERSEN, T.I., BORRESEN, A.L., LI, F.P., GARBER, J. & STRONG, L.C. (1992). Germline mutations of the p53 tumor-suppressor gene in children and young adults with second malignant neoplasms. *New Engl. J. Med.*, **326**, 1309–1315.

MALKIN, D., LI, F.P., STRONG, L.C., FRAUMENI, J.F. Jr, NELSON, C.E., KIM, D.H., KASSEL, J., GRYKA, M.A., BISCHOFF, F.Z., TAINSKY, M.A. & FRIEND, S.H. (1990). Germ line p53 mutations in familial syndrome of breast cancer, sarcomas, and other neoplasms. *Science*, **250**, 1233–1238.

MULVIHILL, J.J., MILLER, R.W. & FRAUMENI, J.F. Jr (1977). *Genetics of Human Cancer*. Raven Press: New York.

NAROD, S.A., FEUNTEUN, J., LYNCH, H.T., WATSON, P., CONWAY, T., LYNCH, J. & LENOIR, G.M. (1991). Familial breast-ovarian cancer locus on chromosome 17q12–q23. *Lancet*, **338**, 82–83.

PELTOMAKI, P., SISTONEN, P., MECKLIN, J.P., PYLKKANEN, L., AALTONEN, L., NORDLING, S., KERE, J., JARVINEN, H., HAMILTON, S.R., PETERSEN, G., KINZLER, K.W., VOGELSTEIN, B. & CHAPELLE, A. (1992). Evidence that the MCC-APC gene region in 5q21 is not the site for susceptibility to hereditary nonpolyposis colorectal carcinoma. *Cancer Res.*, **52**, 4530–4533.

PRITCHARD-JONES, K., FLEMING, S., DAVIDSON, D., BICKMORE, W., PORTEOUS, D., GOSDEN, C., BARD, J., BUCKLER, A., PELLETIER, J., HOUSMAN, D., HEYNINGEN, V.V. & HASTIE, N. (1990). The candidate Wilms' tumour gene is involved in genitourinary development. *Nature*, **346**, 194–197.

SATTIN, R.W., RUBIN, G.L., WEBSTER, L.A., HUEZO, C.M., WINGO, P.A., ORY, H.W. & LAYDE, P.M. (1985). Family history and the risk of breast cancer. *JAMA*, **253**, 1908–1913.

SCHNEIDER, N.R., WILLIAMS, W.R. & CHAGANTI, R.S.K. (1986). Genetic epidemiology of familial aggregation of cancer. *Adv. Cancer Res.*, **47**, 1–35.

STEINBERG, G.D., CARTER, B.S., BEATY, T.H., CHILDS, B. & WALSH, P.C. (1990). Family history and risk of prostate cancer. *Prostate*, **17**, 337–347.

STRONG, L.C., WILLIAMS, W.R. & TAINSKY, M.A. (1992). The Li-Fraumeni syndrome: from clinical epidemiology to molecular genetics. *Am. J. Epidemiol.*, **135**, 190–199.

STRONG, L.C., STINE, M. & NORSTED, T.L. (1987). Cancer in survivors of childhood soft tissue sarcoma and their relatives. *J. Natl Cancer Inst.*, **79**, 1213–1220.

VOGELSTEIN, B., FEARON, E.R., KERN, S.E., HAMILTON, S.R., PREISINGER, A.C., NAKAMURA, Y. & WHITE, R. (1989). Allelotype of colorectal carcinomas. *Science*, **244**, 207–211.

VOGELSTEIN, B., FEARON, E.R., HAMILTON, S.R., KERN, S.E., PREISINGER, A.C., LEPPERT, M., NAKAMURA, Y., WHITE, R., SMITS, A.M. & BOS, J.L. (1988). Genetic alterations during colorectal-tumor development. *New Engl. J. Med.*, **319**, 525–532.

WILLIAMS, W.R. & STRONG, L.C. (1985). Genetic epidemiology of soft tissue sarcomas in children. In *Familial Cancer*, Muller, H.R. & Weber, W. (ed). pp. 151–153. A.G. Karger: Basel, Switzerland.